# Analysis of Pyrrolizidine Alkaloids in Stingless Bee Honey and Identification of a Botanical Source as *Ageratum conyzoides*

**DOI:** 10.3390/toxins16010040

**Published:** 2024-01-12

**Authors:** Natasha L. Hungerford, Norhasnida Zawawi, Tianqi (Evonne) Zhu, Steve J. Carter, Kevin J. Melksham, Mary T. Fletcher

**Affiliations:** 1 Queensland Alliance for Agriculture and Food Innovation (QAAFI), The University of Queensland, Health and Food Sciences Precinct, Coopers Plains, QLD 4108, Australia or n.zawawi@uq.edu.au (N.Z.); mary.fletcher@uq.edu.au (M.T.F.); 2 Faculty of Food Science and Technology, University Putra Malaysia, Serdang 43400, Malaysia; 3 Forensic and Scientific Services, Queensland Health, Coopers Plains, QLD 4108, Australia; steve.carter@health.qld.gov.au (S.J.C.); kevin.melksham@health.qld.gov.au (K.J.M.)

**Keywords:** UHPLC-MS/MS, pyrrolizidine alkaloid, stingless bee honey, Meliponini, *Ageratum conyzoides*

## Abstract

Stingless bee honeys (SBHs) from Australian and Malaysian species were analysed using ultra-high performance liquid chromatography-tandem mass spectrometry (UHPLC-MS/MS) for the presence of pyrrolizidine alkaloids (PAs) and the corresponding *N*-oxides (PANOs) due to the potential for such hepatotoxic alkaloids to contaminate honey as a result of bees foraging on plants containing these alkaloids. Low levels of alkaloids were found in these SBHs when assessed against certified PA standards in targeted analysis. However, certain isomers were identified using untargeted analysis in a subset of honeys of *Heterotrigona itama* which resulted in the identification of a PA weed species (*Ageratum conyzoides*) near the hives. The evaluation of this weed provided a PA profile matching that of the SBH of *H. itama* produced nearby, and included supinine, supinine *N*-oxide (or isomers) and acetylated derivatives. These PAs lacking a hydroxyl group at C7 are thought to be less hepatoxic. However, high levels were also observed in SBH (and in *A. conyzoides*) of a potentially more toxic diester PA corresponding to an echimidine isomer. Intermedine, the C7 hydroxy equivalent of supinine, was also observed. Species differences in nectar collection were evident as the same alkaloids were not identified in SBH of *G. thoracica* from the same location. This study highlights that not all PAs and PANOs are identified using available standards in targeted analyses and confirms the need for producers of all types of honey to be aware of nearby potential PA sources, particularly weeds.

## 1. Introduction

Stingless bee honey (SBH) is the product of native stingless bees, in the tribe Meliponini, which are highly eusocial bees that mainly inhabit tropical and subtropical areas of the world. More than 600 different species of stingless bees have been described, with new species identified each year. With more than 1600 species of bees in Australia, only 12 are stingless bees with most of these occurring in northern regions of Australia including Queensland and northern New South Wales due to the preference for warmer climates. Stingless bees, like other eusocial bees, collect nectar for honey production and can produce more honey than needed for their own survival in warm climates [1]. Australian native stingless bees belong to genera *Austroplebeia* and *Tetragonula* and, like honeybees, are generalists, exploiting a wide range of plant taxa for their floral resources. *Tetragonula carbonaria* and *Tetragonula hockingsi* are two of the main species of stingless bees kept domestically in Queensland. In Malaysia, two stingless bee species, *Heterotrigona itama* and *Geniotrigona thoracica*, are kept in managed hives for honey production [2], with the former being the most common.

Interest in stingless bees and their honey has increased in both Australia and Malaysia due to the popularity of keeping of stingless bees in managed hives, with motivations including commercial products such as honey, propolis and pollination services and biodiversity maintenance. Recent infestations of Varroa mite (*Varroa destructor*) in *Apis mellifera* hives in Australia have further increased interest in Australian native bees, which are not affected by Varroa mites. Stingless bee honey is highly prized and has several unique features, including that it is stored by the bees in cerumen pots, which contribute distinctive phytochemicals to the honey [3]. Stingless bee honey, whilst produced in small volumes, has a unique and sought-after flavour, with interest growing after the identification of the novel disaccharide trehalulose as a low glycemic index (GI) sugar in the honey of five species [4] and there is also interest in SBH medicinal properties [5].

Unfortunately, floral nectar can also contribute other less desirable compounds to honey. Pyrrolizidine alkaloids (PAs) are potentially toxic secondary metabolites of plants and PAs and their corresponding *N*-oxides (PANOs) are produced by an estimated 3% of flowering plants [6] including Asteraceae (Senecioneae tribe), Boraginaceae and Fabaceae (Crotalarieae tribe). PAs consist of a necine base forming esters with one or more necic acids. These alkaloids are potent carcinogens and the double bond between carbons 1 and 2 of the necine base is requisite for their hepatotoxic action. 1,2-Unsaturated PAs/PANOs generally include necine bases of the retronecine, heliotridine or otonecine type.

Numerous studies have identified toxic PAs in honey produced by honeybees (*Apis mellifera*) [7,8,9,10], with PAs transferred from plant pollen or nectar to the hive by the bees. Many of the PA-containing plants are widely distributed weeds (*Crotalaria*, *Echium*, *Senecio*, *Heliotropium* species) with some seemingly sought out by honeybees that can travel an average of 5 km from their hives [11]. With the limited foraging range of stingless bees, (for example *T. carbonaria* has a maximum homing range of 712 m [12]), then PA-containing plants would need to be within close proximity of the hive for there to be any PA contamination of stingless bee products. Notably, however, PAs have been reported in the propolis of Brazilian *Scaptotrigona postica* [13,14,15,16] but not in stingless bee honey. PAs were previously searched for in the pot-pollen of *T. carbonaria*, *T. hockingsi* and *Austroplebeia australis* in the mass range *m*/*z* 398.2184 –*m*/*z* 496.3402, but no PAs were detected [17], using HPLC combined with a diode array detector and Orbitrap high-resolution mass spectrometry.

International concerns have heightened regarding dietary exposure to PAs, which can variously contaminate the food chain by the direct consumption of PA-containing plants, or the consumption of plant-derived products inadvertently contaminated with PA-containing plants, e.g., from weeds co-harvested with raw materials. The collection of nectar and pollen from PA-containing plants by bees can lead to the contamination of bee products such as honey. In Europe, maximum levels of PAs in herbal products and teas are now legislated [18].

Many analytical methods rely on targeted liquid chromatography-tandem mass spectrometry (LC-MS/MS) analyses requiring the acquisition of reference standards. Of the >600 known PAs, there are limited PAs commercially available as standards, with added complexity due to the numerous potential stereoisomers, some with identical mass spectra. Our previously reported approach utilising Orbitrap high-resolution accurate mass (HRAM) spectrometry enables the characterisation of targeted, untargeted and suspected PAs, including isomers, by providing full scan data as well as suitable MS^2^ spectra for structural elucidation [7,19] and comparisons with plants reported to contain PAs. Such an approach has recently been expanded to a library of 118 PAs and PANOs for studies across various food matrices [20,21]. To date, however, no such PA study has been reported for SBH.

In this study, we aimed to apply our Orbitrap approach to identify PAs in honey from four stingless bee species, *T. carbonaria* and *T. hockingsi* in Australia and *Heterotrigona itama* and *Geniotrigona thoracica* in Malaysia. This analysis reveals low levels of stingless bee honey contamination by PAs, with the alkaloid profile of a subset of Malaysian SBH consistent with a PA-containing weed species growing within the close vicinity of certain hives. Given that the interest in stingless bee honey in Malaysia and Australia is growing, the identification of plant sources that may result in PA contamination is crucial to reducing the human consumption of these toxins through the precautionary adjustment of hive placement or weed removal.

## 2. Results and Discussion

### 2.1. Quantitation of Stingless Bee Honey Alkaloids against Pyrrolizidine Alkaloid Standards

**Figure 1:**
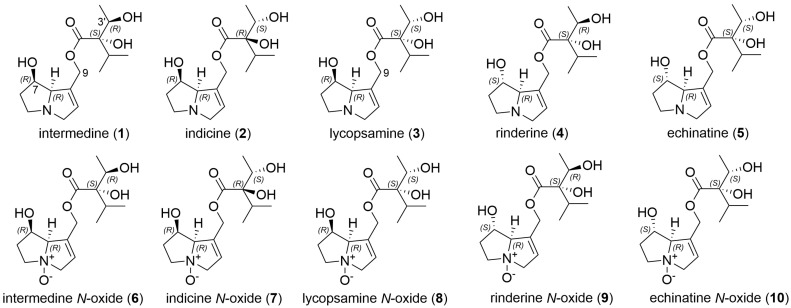
Examples of a known set of isomeric pyrrolizidine alkaloids identified from various plants including intermedine (**1**), indicine (**2**), lycospamine (**3**), rinderine (**4**) and echinatine (**5**) and the corresponding *N*-oxides (**6**)–(**10**).

Stingless bee honey samples were collected directly from managed hives in Queensland (Australia) and Malaysia and the previously validated method for determining pyrrolizidine alkaloid levels in honeybee honey was used [7]. PA levels were quantitated using high-resolution accurate mass (HRAM) UHPLC-MS/MS using certified PAs as 30 external standards, and targeted analysis with a measurement of the precursor ion intensity relative to the standard curves (see Section 3.7). PA identification using UHPLC-MS/MS involved a comparison of the standards for retention time and precursor ion identification, with verification by MS/MS fragments present together with their relative abundance. As previously observed, in some instances, isomeric PAs display identical mass spectra and differentiation requires separation by retention time, which, in our previous study, was achieved for 30 certified PA standards, except for co-eluting indicine *N*-oxide and intermedine *N*-oxide. In particular, the separation of intermedine (**1**), indicine (**2**) and lycopsamine (**3**) required chromatography at 5 °C [7]. Attempts to separate and quantitate all five isomeric PAs with *m*/*z* 300 including rinderine (**4**) and echinatine (**5**) have been published [22]. The structures of these pyrrolizidine alkaloids with *m*/*z* 300 and the corresponding *N*-oxides (*m*/*z* 316) (**6**–**10**) are shown in Figure 1. Without all PAs available as standards, unknowns can be tentatively identified (untargeted identification).

The current survey of PAs in stingless bee honey from Australia and Malaysia found seven PAs/PANOs above the Limit of Reporting (LOR) (Table 1) in comparison with the 30 PA standards. These included indicine, intermedine, indicine *N*-oxide/intermedine *N*-oxide (the *N*-oxides of these co-eluted), lycopsamine, lycopsamine *N*-oxide and jacobine. Using the targeted PA standards, PAs determined in each sample occurred at a level above LOR in five out of twenty-one Queensland samples only, with a maximum level of 85 ng/g observed for lycopsamine in *T. carbonaria* SBH and all PAs below LOR for *T. hockingsi* samples. By the same method, in Malaysian honey samples, PAs occurred at a level above LOR in six out of fifteen samples with a maximum level of 391 ng/g for co-eluting indicine *N*-oxide/intermedine *N*-oxide in a *Heterotrigona itama* SBH and a maximum level of 297 ng/g for intermedine, with one sample only of *Geniotrigona thoracica* revealing low levels of PAs above LOR. These levels observed in stingless bee honey were much lower than our previous survey of commercial honeybee honey, which found levels of lycopsamine up to 3100 ng/g [7]. This is an interesting observation given that some of the stingless bee honeys were sourced from locations in suburban Brisbane, not far from that of commercial honeybee honey samples reported in this previous study.

### 2.2. Untargeted Analysis of Novel PAs in Stingless Bee Honey Samples

**Figure 2:**
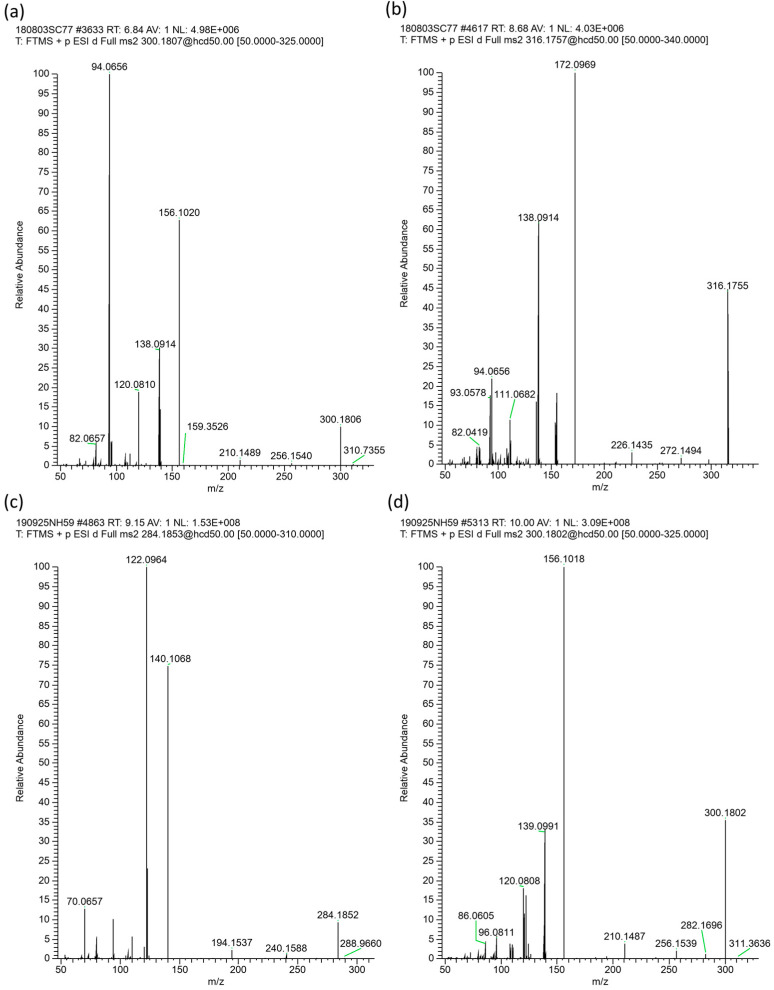
MS/MS spectra for (**a**) lycopsamine (**b**) lycopsamine *N*-oxide pyrrolizidine alkaloid standards and (**c**) precursor ion MH^+^ 284.1852 (**d**) precursor ion MH^+^ 300.1802 in stingless bee honey sample HI-7.

The higher PA-containing SBH samples were a subset from *Heterotrigona itama* (HI-6–HI-10), with the highest levels from HI-6 and HI-7 sourced from hives co-located at the same property. Closer inspection of the full-scan and dd-MS^2^ scans (Section 3.8) of these honey samples revealed the presence of PAs/PANOs that were not targeted by the standards. Isomeric peaks that differed in retention time from the standards could be observed via the extracted ion chromatograms. The largest of these peaks was isomeric with intermedine/lycopsamine with MH^+^ of *m*/*z* 300.1802 but with a substantially different retention time of 10.1 min. However, the fragmentation pattern was different, and instead of the base peak of *m*/*z* 94.0656 usually observed for lycopsamine (**3**) on our system (see Figure 2a) and its isomers indicine (**2**) and intermedine (**1**), the unknown PA at 10.1 min displayed a base peak of *m*/*z* 156.1018 (Figure 2d). To assess whether this PA was present as the *N*-oxide form, the honey sample was treated with acidified zinc, known to reduce *N*-oxides to the corresponding free amine. The treatment of the honey sample with acidified zinc resulted in reduction, with the peak at *m*/*z* 300.1802 converted to MH^+^ of *m*/*z* 284.1856 (9.2 min), with two main MS/MS fragments at *m*/*z* 140.1070 and *m*/*z* 122.0965 (Figure 2c). This reduction suggested that the unknown PA at *m*/*z* 300.1802 was an *N*-oxide, but without the typical base peak at *m*/*z* 172.0969 observed for lycopsamine-type *N*-oxides (as shown in Figure 2b). Instead, the *N*-oxide base peak was observed at *m*/*z* 156.1018, 15.9951 amu (equivalent to an oxygen) less than *m*/*z* 172.0969.

**Figure 3:**
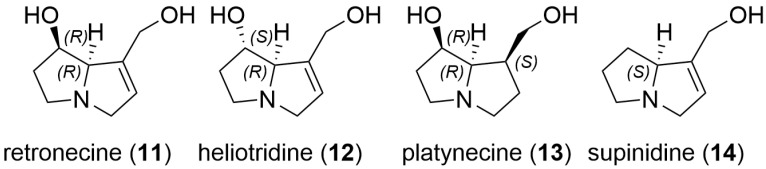
A comparison of pyrrolizidine bases including 1,2-unsaturated bases retronecine (**11**) and heliotridine (**12**), and those either lacking 1,2-unsaturation (eg platynecine (**13**)) or 7-hydroxy substitution (eg supinidine (**14**)).

**Figure 4:**
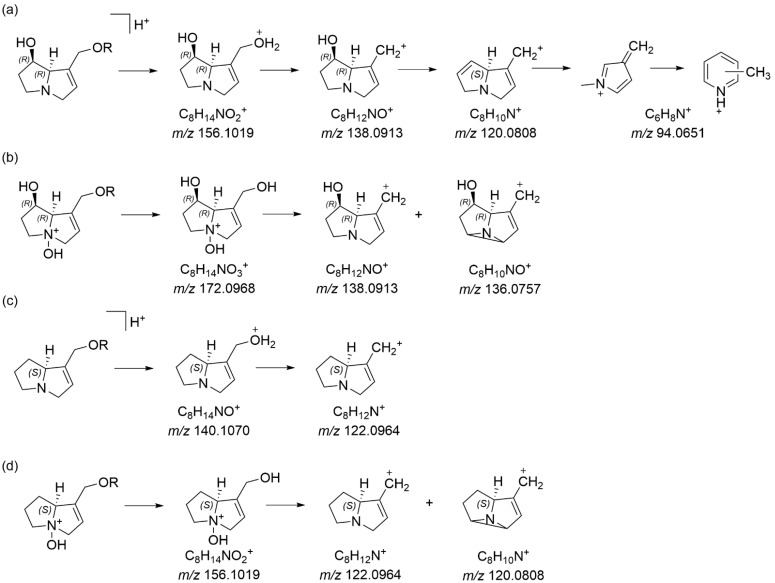
Fragments typical of (**a**) retronecine (and heliotridine (**12**), structures not shown) monoesters, (**b**) retronecine *N*-oxide (and heliotridine *N*-oxide, structures not shown) monoesters, (**c**) supinidine esters and (**d**) supinidine *N*-oxide esters (R = necic acid) [21,28,29], with fragment masses observed via Orbitrap HRAM spectrometry in the current study.

**Figure 5:**
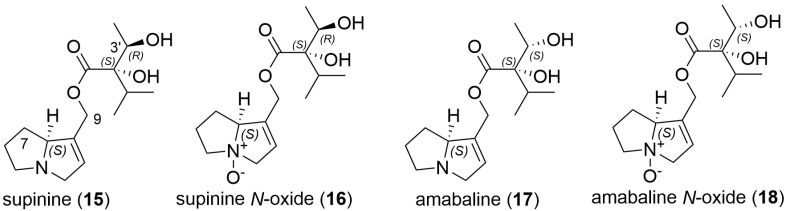
Structures of example supinidine monoester pyrrolizidine alkaloids.

Further examination of the honey sample prior to zinc reduction also showed the presence of the compound with MH^+^ 284.1856, which was therefore also naturally present in this SBH. Whilst fragment ions of 140.1070 and 122.0965 can be indicative of a platynecine base in the pyrrolizidine alkaloid, the absence of characteristic peaks at *m*/*z* 174.1125 in the *N*-oxide and a *m*/*z* 158.1176 peak for the reduced compound [21] suggested that this was not a PA with a platynecine base. Instead, the observed fragments suggested the absence of a 7-hydroxy group, and the presence of a supinidine type base (explaining the difference of 16 amu). A comparison of retronecine-, heliotridine-, platynecine- and supinidine-type pyrrolizidine bases (**11**–**14**) is shown in Figure 3. The expected fragments from monoesters of retronecine- and supinidine-type pyrrolizidine alkaloids are shown in Figure 4a–d. The observed fragments of *m*/*z* 140.1070 and *m*/*z* 122.0965 (base peak) are explained by the proposed structures in Figure 4c and the fragments observed for the *N*-oxide, including a base peak of *m*/*z* 156.1019, are shown in Figure 4d. Hence, the unknown PA and PANO in Malaysian stingless bee honey were proposed to be supinine (**15**, Figure 5) (calculated MH^+^ of *m*/*z* 284.1856) or a stereoisomer and the corresponding *N*-oxide (**16**, Figure 5) (calculated MH^+^ of *m*/*z* 300.1805). A minor isomer of the *N*-oxide (MH^+^ of *m*/*z* 300.1804), displaying a very similar fragmentation pattern to (**16**), was observed at 10.6 min and proposed to be amabaline *N*-oxide (**18** or isomer). However, the reduced form (potentially amabaline (**17**) (or isomer)) was not observed at any significant level. The base peak of *m*/*z* 122 has been previously described for supinine [23] and amabaline by GC-MS [24] and UHPLC-MS/MS [21] and they have been identified in numerous species [25], isolated from *Eupatorium laevigatum* [26], with amabaline and further isomers isolated from *Heliotropium spathulatum* [27]. The assignment of chromatographic peaks to the stereochemistry of supinine versus amabaline (and their *N*-oxides) is informed by the stereochemistry and predominance of other PAs identified as also containing the necic acid, (+)-trachelanthic acid (Section 2.3 and Section 2.4). This inferred stereochemistry is based on comparisons with standards and plants with known PAs.

The need to search for PA-containing plants in the vicinity of these hives was recognised. The detection of alkaloids in such a plant would provide a distinctive PA fingerprint to compare with stingless bee honey samples in an attempt to ascertain the floral source of the PAs and PANOs. The weed species, *Ageratum conyzoides* L. (Herbarium Faculty of Forestry, Universiti Putra Malaysia, Voucher No. H080) and *Chromolaena odorata* L., were soon found and identified after a survey of the hive surroundings for HI-6 and HI-7. Plant materials were separated into plant parts, freeze-dried and extracted, with the extracts being sent to Australia for analysis using HRAM UHPLC-MS/MS.

### 2.3. Survey of Alkaloids in Chromolaena odorata L.

*Chromolaena odorata* (L.) R.M. King and H. Rob. (Asteraceae) is an invasive plant in the tropics and subtropics [30,31] and was sourced from the vicinity of hives HI-6 and HI-7. A brief examination using targeted/untargeted analysis of the HRAM data of *Chromolaena odorata* flowers, after SCX-SPE clean-up, enabled the tentative identification of PAs with MH^+^ of *m*/*z* 300.1805 (two peaks at RT 6.71 min and 7.25 min) and the corresponding *N*-oxides at MH^+^ of *m*/*z* 316.1755 (two peaks at RT 8.36 min and 8.67 min). The peaks at 6.71 min and 8.67 min matched the retention time and fragmentation pattern of intermedine (**1**) and intermedine *N*-oxide (**6**), respectively (Table 2). Whilst the peak at 7.25 min matched the retention time of lycopsamine, the base peak was now *m*/*z* 138.0915 rather than the *m*/*z* 94.0656 observed for lycopsamine. In addition, the corresponding *N*-oxide peak did not match the retention time of lycopsamine *N*-oxide standard (RT 9.15 min). Instead, the observed peaks at 7.25 min and 8.36 min are proposed to be rinderine and rinderine *N*-oxide (Table 2), as observed previously in this species [32,33]. The observed mass spectra matched those reported by [23] for rinderine (**4**) and rinderine *N*-oxide (**9**). No peaks were identified corresponding to the supinidine-type pyrrolizidine bases as identified in our stingless bee honey samples of interest, so the plant was not considered to be the source of the PAs in these honey samples.

### 2.4. Pyrrolizidine Alkaloids Determined in Weed Ageratum conyzoides L.

*Ageratum conyzoides* L. is the most studied plant in the genus Ageratum and is a widespread weed in many tropical parts of the world [34,35]. The tentative identification of alkaloids in *A. conyzoides* by HRAM UHPLC-MS/MS in this study enabled a comparison with the PA profiles of stingless bee honey samples to gather evidence that this plant species is the source of PAs in certain Malaysian honey samples.

The literature tells a confusing story of the alkaloids in *A. conyzoides*. A study of *A conyzoides* collected in Kenya revealed the presence of two PAs, lycopsamine and echinatine, as determined by MS and NMR [36]. Another study [37] of *A. conyzoides*-derived tea from Brazil identified lycopsamine, dihydrolycopsamine, acetyllycopsamine, lycopsamine *N*-oxide, dihydrolycopsamine *N*-oxide and acetyl-lycopsamine *N*-oxide. Collected *A. conyzoides* from Hawaii indicated lycopsamine and 3′-acetyllycopsamine and their *N*-oxides as well as evidence of some other likely dehydro-PAs [38]. Mixed parts of *A. conyzoides* from Missouri (USA) were found to contain lycopsamine (0.2 ± 0.4 µg/g), present together with its *N*-oxide and other minor amounts of dihydrolycopsamine and *N*-oxide and echinatine [39]. By contrast, a study correlating PAs in weeds and tea in China proposed high levels of intermedine and intermedine *N*-oxide and heliotrine and its *N*-oxide in *A. conyzoides* using a limited set of 15 PA alkaloid standards [40]. Given the high number of isomers of PAs, it is possible that some misidentification of PAs has occurred and full fragmentation pattern details are not always documented. Alternatively, it is possible that different chemotypes of this same species occur in different regions. Regardless, the potential PA toxicity of this plant remains a concern, and fatal poisonings of people in the Tigray region of Ethiopia have been attributed to the contamination of millet with *A. conyzoides* [41].

**Figure 6:**
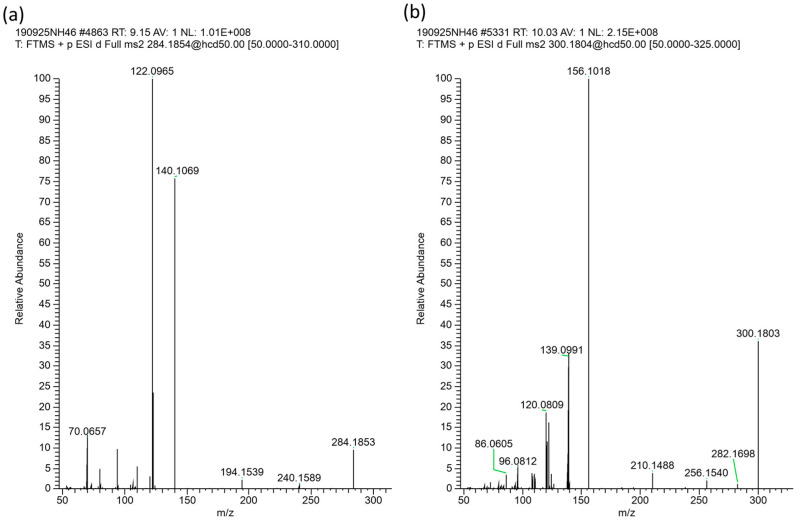
High resolution accurate mass spectra for (**a**) *Ageratum conyzoides* peak at 9.15 min tentatively corresponding to supinine (or isomer) MS/MS, (**b**) *Ageratum conyzoides* peak at 10.03 min tentatively corresponding to supinine *N*-oxide (or isomer) MS/MS, matching the mass spectra observed in Malaysian stingless bee honey HI-7 in Figure 2c,d.

In the present study, *A. conyzoides* plant material was sourced from the vicinity of Malaysian hives (HI-6 and HI-7) and it was hoped that it would contain the tentatively identified supinine and supinine *N*-oxide in stingless bee honey (HI-6–HI-10). After extraction and SCX-SPE clean-up, PAs were identified by targeted analysis and, in addition, the untargeted analysis of the HRAM data of *A. conyzoides* flowers enabled the tentative identification of further pyrrolizidine alkaloids (Table 3). The largest peaks of supinine (**15**) and supinine *N*-oxide (**16**) in *A. conyzoides* flowers provided MS/MS spectra (Figure 6a,b, respectively), which matched those observed in honey samples HI-6–HI-10 and those shown for HI-7 in (Figure 2c,d), so further analysis of this plant was undertaken. An analysis of Zn reduced flower extracts revealed the conversion of *N*-oxide peaks to the corresponding reduced free alkaloid form of each PA.

**Figure 7:**
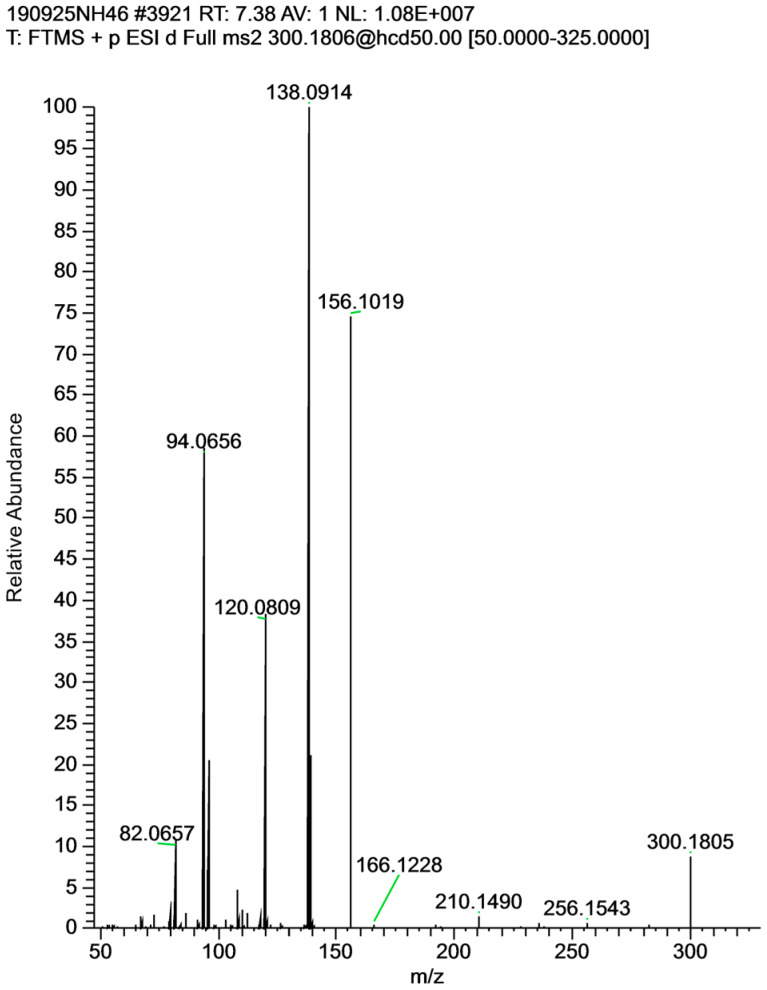
HRAM MS/MS spectrum for chromatography peak with same retention time (RT 7.38 min) as lycopsamine in *A. conyzoides* flowers, showing base peak of *m*/*z* 138.0914, and tentatively assigned as rinderine (**4**).

The details of tentatively identified PA molecular formulas, calculated MH^+^ values and observed MS/MS spectra are included in Table 3. Targeted analysis, comparing with the PA standards, suggested the presence of intermedine and lycopsamine (~2:1) and their *N*-oxides. The peak observed at the retention time of the lycopsamine standard (7.4 min, MH^+^ of *m*/*z* 300.1805) did not exhibit the same relative peak heights usually observed for lycopsamine with a base peak of *m*/*z* 138.0914 (compare Figure 7 with Figure 2a for lycopsamine). Isomers epimeric at C7, including rinderine (**4**) and echinatine (**5**) (Figure 1), show base peaks of *m*/*z* 138.0914 and so the 7.4 min peak is proposed to be a mixture of co-eluting lycopsamine and rinderine. The retention time and MS/MS matched those observed for rinderine in *C. odorata*. Previously, echinatine and lycopsamine were reported in *A. conyzoides*, based on MS and NMR spectra of isolated alkaloids. Another study of *A. conyzoides*, noted the presence of a number of PA isomers [39], which is consistent with our observations herein. NMR studies of all four isomeric PAs (**1**)–(**4**) [42] (in deutero-chloroform, CDCl_3_) provided a good correlation with the spectra of echinatine and lycopsamine reported by Wiedenfeld [36] (in CDCl_3_/deuterated dimethylsulfoxide (d_6_-DMSO)) except that the chemical shifts for H9 suggest the isomeric pair as reported from *A. conyzoides* could equally well be rinderine and intermedine, considering the differing NMR solvents used.

**Figure 8:**
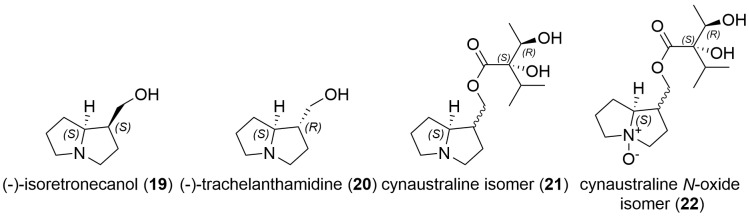
Structures of further pyrrolizidine bases together with isomers of cynaustraline and *N*-oxide (stereochemistry unknown) found in *A. conyzoides* and certain stingless bee honeys.

An observed acetylated intermedine *N*-oxide (MH^+^ of *m*/*z* 358.1859) was determined to be acetylated at 3′ hydroxyl, giving 3′-*O*-acetylintermedine *N*-oxide (or stereoisomers) based on the absence of a base peak at *m*/*z* 214.1074, which was observed as dominant for the alternative 7-*O*-acetyl compounds due to the stability of the non-allylic C7 esters under mass spectral conditions [43,44]. The intermedine stereochemistry was presumed based on this being the predominant isomer. Further precursor peaks observed included an MH^+^ of 238.1442 and *m*/*z* 238.1439 and an MH^+^ of 254.1386 and *m*/*z* 254.1385, which were either angelyl or tiglyl esters of retronecine and the *N*-oxides, respectively. The assignments of 9- versus 7-*O*-angelylretronecine *N*-oxide (or tiglyl) esters were based on previous Orbitrap HRAM spectra [21] with base peaks of *m*/*z* 106.0655 observed in the latter *N*-oxides. A further chromatogram peak at 13.8 min was observed to be isomeric with echimidine C_20_H_31_NO_7_ + H^+^ observed to be MH^+^ of *m*/*z* 398.2173 with a base peak observed at *m*/*z* 120.0810, and the corresponding *N*-oxide (MH^+^ of *m*/*z* 414.2122) observed at 15.2 min. Compounds containing the saturated trachelanthamidine or isoretronecanol-type base (Figure 8), which lack the 7-hydroxy group, such as cynaustraline isomers, were observed with an MH^+^ of *m*/*z* 286.2012 and a base peak *m*/*z* 142.1227 whilst the corresponding *N*-oxide displayed an MH^+^ of *m*/*z* 302.1961 and a base peak *m*/*z* 158.1175 (refer to Table 3).

### 2.5. Honey PA Profiles Linked to A. conyzoides

**Figure 9:**
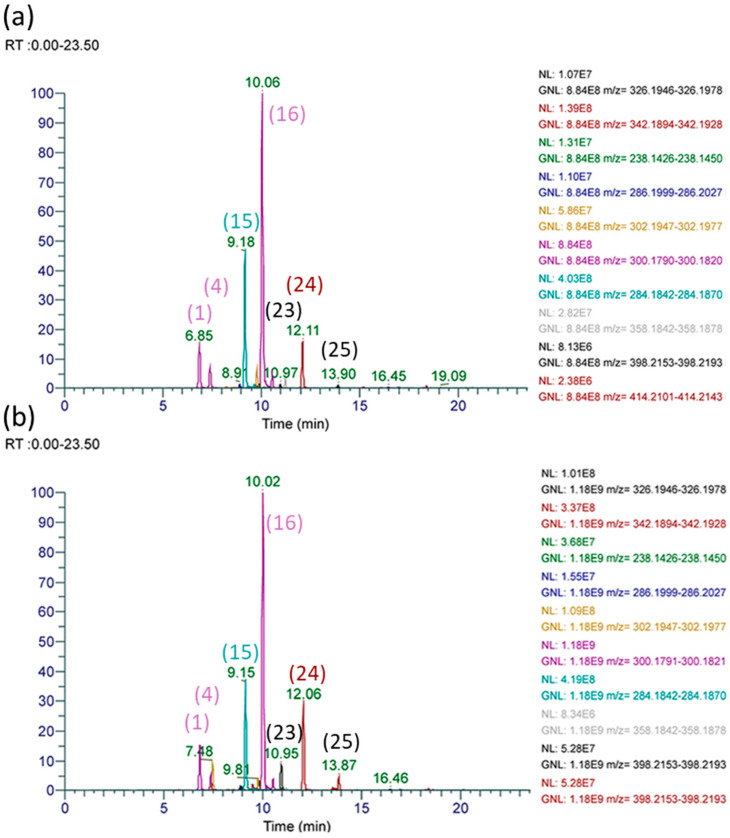
HRAM UHPLC-MS/MS chromatograms of prominent pyrrolizidine alkaloids of (**a**) extracted *A. conyzoides* flowers and (**b**) extracted stingless bee honey sample HI-7 showing the major peaks corresponding to supinine *N*-oxide (**16**) and supinine (**15**) and 3′-*O*-acetylsupinine *N*-oxide (**24**, or isomers) and echimidine isomer (**25**).

The profile of alkaloids identified in *Ageratum conyzoides* provided a distinctive PA fingerprint to compare with alkaloids observed in certain Malaysian stingless bee honeys. The chromatograms in Figure 9, which compare prominent alkaloids in the extracted *A. conyzoides* flowers with those observed in the SBH, provides a clear indication that this weed species is being used as the source of nectar for honey by stingless bees of *H. itama*. Despite the very close vicinity of hives of *G. thoracica*, the same alkaloids were not observed in the honey of this species, collected on the same date, suggesting species differences in sourcing nectar for honey production that can impact PAs in honey. All of the PAs identified in *A. conyzoides*, as listed in Table 3, were also found in stingless bee honey of *H. itama* (HI-7).

A comparison of the intermedine *N*-oxide isomer peaks (*m*/*z* 316.1755) in *A. conyzoides* and HI-7 honey showed four peaks with matching retention times, with very similar ratios in peak heights, all with a base peak of *m*/*z* 172.0968. The retention time comparisons with the standards suggested the presence of lycopsamine *N*-oxide (RT 9.35 min, minor) and intermedine *N*-oxide (RT 8.84 min). Another peak was assigned to rinderine *N*-oxide (RT 8.54 min) and a further minor peak (RT 9.12 min) was also observed.

In addition to quantitation using the 30 PA standards (Table S1), the quantities of the PAs listed in Table 4 in SBH were estimated based on the further development of the method post-acquisition. As detailed in Table 4, precursor ions and product ions were calculated after interrogation of the HRAM data provided by the Q Exactive mass spectrometer, including the full-scan data with data-dependent dd-MS^2^, giving good quality MS^2^ spectra. Despite the use of diagnostic fragment ions for the different necine base types, (Figure 3, Figure 4 and Figure 8) [28,29], the complete identification of PAs was not possible due to the large number of isomers and the limited numbers of PAs available as standards. Tentatively identified PAs were added to the compound library, with retention time, molecular formula, precursor ions and most abundant product ions, with a mass tolerance of 5 ppm, and a minimum of three product ions required for identification. The levels of the observed PAs were estimated using the calibration curves of lycopsamine, lycopsamine *N*-oxide, echimidine or echimidine *N*-oxide and, in some cases, extrapolations undertaken to gain an estimate of levels.

**Figure 10:**
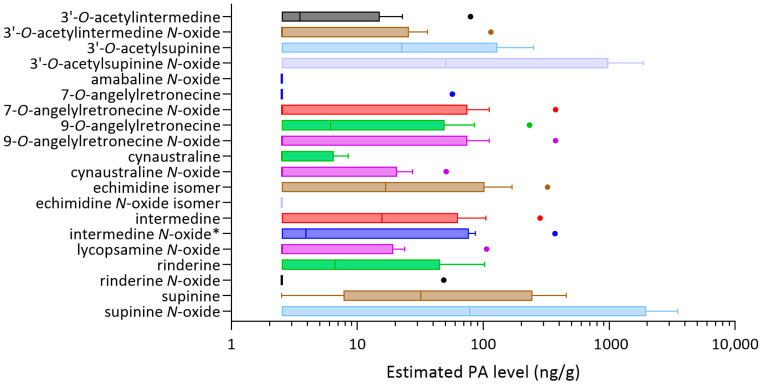
Tukey box plots showing estimated levels of each pyrrolizidine alkaloids for honey analysed from *H. itama*. Values below LOR were substituted for LOR/2. * Intermedine *N*-oxide co-eluted with indicine *N*-oxide.

The only SBH samples that contained significant levels of PAs were a subset of honeys of *H. itama*. Data obtained for all *H. itama* honey samples studied are shown in Figure 10, combining the results obtained for PAs with and without PA standards available for comparison. Any data below the LOR were substituted for LOR/2. The highest levels were estimated to be for supinine (**15**) and 3′-*O*-acetylsupinine (**23**) and their corresponding *N*-oxides ((**16**) and (**24**), or isomers).

### 2.6. Health Implications of Different PA Types

The highest levels of PAs identified in stingless bee honey corresponded to PAs lacking the 7-hydroxy group (Figure 10). 1,2-Unsaturated PAs are well known hepatotoxins of botanical origin. Conversion to pyrrolic metabolites is a requirement for their toxic effect via reactions with cellular proteins and DNA, forming adducts at the C7 and/or C9 positions, leading to hepatotoxicity, genotoxicity and tumorigenicity [45,46,47]. Most necine bases are esterified with a variety of necic acids at the 1-hydroxymethyl and/or 7-hydroxy groups forming monoester PAs, open-chain diester PAs or macrocyclic diester PAs. Monoesters have been reported to be less toxic than the more toxic diesters and macrocyclic esters [48]. Supinidine-type esters lacking a C7 substituent, such as those identified in this study, were found to be only weakly hepatotoxic [49] with less data reported. Such low level hepatoxicity is reassuring in this case, but high levels were also observed in the SBH (and in *A. conyzoides*) of a potentially more toxic diester PA corresponding to an echimidine isomer, and of intermedine, the C7 hydroxy equivalent of supinine. The cumulative toxicity of PAs with 1,2-unsaturation has been demonstrated [45] and the European Food Safety Authority (EFSA) concluded there was a possible human health concern related to chronic cumulative exposure to PA-contaminated food products [50]. Maximum levels of PAs in teas and herbal products, including pollen products, are now legislated in Europe [18], with limits set for the sum of 21 PAs, together with 14 PAs known to co-elute, such that the PAs identified (individually or separately) by this method shall be quantified and included in the sum.

This study highlights the importance of the monitoring of PAs in bee products and of the location of hives with relation to the presence of PA-containing plants. This is shown to be the case for stingless bee honey hives, as previously observed for honeybee honey hives [19,51,52]. The types and levels of PAs present in both types of honey are linked to the location and types of flowering plants with PAs. The siting of hives to reduce PA exposure within SBH is therefore similarly recommended. Species preference for nectar sources may also impact PA levels in honey, as observed for *H. itama* versus *G. thoracica* hives both located near *A. conyzoides*. The vast variety of PAs (over 600 known compounds) and the limited number of these available as reference standards means that targeted UHPLC-MS/MS methods are limited as to which PAs they can detect and monitor in foods. This importantly highlights the need for methods that can both identify and expand the set of PAs monitored in honey and other products.

## 3. Materials and Methods

### 3.1. Chemical and Solvents

Certified standards of 30 PAs were purchased from Phytolab GmbH & Co. KG (Vestenbergsgreuth, Germany, purity > 89%) and were used in an HRAM UHPLC-MS/MS targeted screen, including echimidine, erucifoline, europine, heliotrine, indicine, intermedine, jacobine, lasiocarpine, lycopsamine, monocrotaline, retrorsine, senecionine, seneciphylline, senecivernine, and their corresponding *N*-oxides, as well as senkirkine and trichodesmine. All water used was Milli-Q purified (Merck Millipore, Darmstadt, Germany). Purity of all other solvents/chemicals were of analytical or HPLC grade.

### 3.2. Honey Samples

Thirty-six stingless bee honey samples, also employed in another study [2], were collected from different states in Malaysia and different regions in Queensland (Australia), as shown in Table 5. Samples produced by four different stingless bee species (refer to Table 5) were freshly collected directly from beehives (without any processing treatment) between January and June 2018. Before analysis, all honey samples were transferred and stored refrigerated at 4 °C in new, sterile plastic bottles.

### 3.3. Stingless Bee Honey Samples for Quantitation against PA Standards

Honey samples (1 g (batch 1) or 2 g (batch 2), triplicates or duplicates, respectively) were treated with aqueous H_2_SO_4_ (0.05 M, 10 mL), centrifuged and the supernatant applied to preconditioned (with methanol (5 mL), then water (5 mL), then 0.05 M H_2_SO_4_ (10 mL)) Bond Elut 100 mg LRC-SCX SPE cartridges (Agilent Technologies, Folsom, CA, USA). Loaded SPE cartridges were washed with water (10 mL) and methanol (10 mL), and then PAs were eluted with 3% ammonia in methanol (3 mL). The obtained samples were evaporated under nitrogen, then reconstituted in 5% methanol/water (1.0 mL (batch 1) or 0.3 mL (batch 2) and filtered (0.2 µm) for HRAM UHPLC-MS/MS analysis. Zn reduction was conducted subsequently by adding 0.05 M H_2_SO_4_ and Zn dust (35 mg). The sample was shaken overnight and filtered then applied to LRC-SCX SPE, as previously.

### 3.4. Stingless Bee Honey Samples for Comparison with Malaysian Plants

Honey samples (2 g) were prepared as in Section 3.3 and reconstituted in 5% methanol in water (0.3 mL) and filtered (0.2 µm) for HRAM UHPLC-MS/MS analysis. Zn reduction was not performed.

### 3.5. Honey Method Validation

The method had been validated in honeybee honey for the 30 PA standards as described in our previous study of *A. mellifera* honeybee honey [7], using blank *A. mellifera* honey and based on values for 10 spiked samples, which gave limits of reporting (LOR) of 5 ng/g for individual PAs, which corresponded to 5 ng/g SBH (batch 1) and 0.75 ng/g SBH (batch 2) determined by sample mass and dilution (refer to Table 5 for individual LOR for each SBH). For some samples (batch 2), the sample taken was increased to 2 g and the reconstitution volume was decreased from 1 mL to 0.3 mL. This meant that, for these samples (as shown in Table 5), LORs could be reduced from 5 ng/g to 0.75 ng/g.

### 3.6. Malaysian Plant Alkaloid Extraction

#### 3.6.1. Plant Sources

*Ageratum conyzoides* L. was collected near hive sources for honeys HI-6 and HI-7 and identified by the Herbarium Faculty of Forestry, Universiti Putra Malaysia (Voucher No. H080). Plants were collected and separated into flower, stem, root and leaf components, then freeze-dried. Dried samples (0.25–1 g) were extracted with methanol (10 mL/g), filtered then concentrated under reduced pressure and sent to Australia for PA analysis.

#### 3.6.2. Plant Extraction and Zinc Reduction

Separate *A. conyzoides* stem, root and leaf dried extracts were weighed (0.23–1.01 g), treated with 0.05 M H_2_SO_4_ (10 mL) and vortexed (20 s), ultrasonicated (5 min) and shaken (10 min). Samples from each (2 mL) were shaken with Zn dust (200 mg) for 2 h. Samples were centrifuged (3900 rpm, 10 min), and for each unreduced and Zn-reduced sample, a 0.1 mL portion of the supernatant was loaded onto preconditioned Agilent Bond Elut LRC-SCX SPE cartridges (500 mg). Cartridges were washed with water (10 mL), then methanol (10 mL), and PAs were eluted (3% ammonia in methanol, 10 mL). The latter solution was evaporated to dryness using nitrogen and then redissolved in 5% methanol in water (1 mL) ready for analysis by UHPLC-MS/MS.

### 3.7. HRAM LC-MS/MS Analysis Using PA Standards

The instrument method was reported in full previously [7], pairing a Vanquish UHPLC with a Q Exactive Orbitrap HRAM spectrometer (Thermo Fisher Scientific, Bremen, Germany). In brief, chromatographic separation of PAs used an analytical Kinetex XB-C18 column (100 × 2.1 mm, 2.6 µm, 100 Å) maintained at 5 °C. The binary solvent system included solvent A consisting of water with 5 mM ammonium formate/0.1% formic acid and solvent B of 95% methanol/water (*v*/*v*) with 5 mM ammonium formate/0.1% formic acid. The flow rate was 0.3 mL/min with solvent B held at 5% (0 to 3 min) then linear gradients of B from 5–50% (3 to 15 min), 50–80% (15 to 18.5 min), 80–100% (18.5 to 19 min). Then, 100% B was held for 30 s, before changing from 100–5% over 6 s, then held, stopping at 23.5 min. Tracefinder 4.1 (Thermo Fisher Scientific) was employed for instrument control, data acquisition and analysis. Alkaloid detection utilised electrospray ionisation (ESI) in positive mode. Full scan MS analysis was combined with data dependent dd-MS^2^ acquisition mode. Full scan resolution was 70,000 FWHM (at *m*/*z* 200), with an AGC target of 1.00 × 10^6^. The scan range was *m*/*z* 75–1125 and maximum injection time of 10 ms and dd-MS^2^ employed a resolution of 17,500, and an AGC target of 1.00 × 10^6^ with maximum injection time of 50 ms. A *m*/*z* 1.0 isolation window and normalized collision energy (nce) of 50% were used. Dynamic exclusion in data-dependent scans was set to 3 s. The TopN parameter was 5 for each dd-MS^2^ scan event.

Quantitation of PAs in honey and plant materials used the 30 certified PA standards, with calibration curves constructed from solutions of 5, 10, 20, 50, 100 and 200 ng/g (injected in duplicate/triplicate). Squared correlation coefficients (R^2^) were in the range of 0.9877–0.9998. Honey or plant extracts were analysed using HRAM UHPLC-MS/MS and PAs and their *N*-oxides were determined by matching retention times with the standard, identifying precursor ions (M + H^+^) and confirming detection with product ions, as detailed in Table S1, and used previously [7]. Elemental compositions were verified using the high-resolution accurate mass data.

### 3.8. HRAM UHPLC-MS/MS Analysis for the Identification of PAs Other Than the Targeted PA Standards

Further PAs were identified through use of the HRAM data provided by the Q Exactive mass spectrometer in full-scan and dd-MS^2^ mode, enabling elemental composition of parent and fragment ions to be determined (Table 4) and enabling matchup between plant and stingless bee honey PAs.

Tentative isomers and corresponding *N*-oxides were indirectly estimated by using the calibration curve of lycopsamine and lycopsamine *N*-oxide, respectively, and assuming the same response and LORs, with the exception of the echimidine isomer and echimidine *N*-oxide isomer, which employed the calibration curve of echimidine and echimidine *N*-oxide to estimate concentrations.

## Figures and Tables

**Table 1:** Pyrrolizidine alkaloid data obtained using PA standards via Orbitrap analysis of stingless bee honey from Malaysian species (*G. thoracica* and *H. itama*) and Australian species (*T. carbonaria* and *T. hockingsi*).

Compound	Malaysian Stingless Bee Honeys						Australian Stingless Bee Honeys					
	*Geniotrigona thoracica* (*n* = 5)			*Heterotrigona itama* (*n* = 10)			*Tetragonula carbonaria* (*n* = 11)			*Tetragonula hockingsi* (*n* = 10)		
	Mean (ng/g)	SD (ng/g)	Range (ng/g)	Mean (ng/g)	SD (ng/g)	Range (ng/g)	Mean (ng/g)	SD (ng/g)	Range (ng/g)	Mean (ng/g)	SD (ng/g)	Range (ng/g)
indicine	2.4	0.6	<LOR—2.8	6.4	11.8	<LOR—42.1	1.4	1.2	<LOR—3.2	<LOR		
indicine *N*-oxide/ intermedine *N*-oxide ^a^	<LOR			62	110	<LOR—391	<LOR	1.1		<LOR		
intermedine			1.9–3.8	52	85	<LOR—297	<LOR	1.0		<LOR		
jacobine	<LOR			<LOR			<LOR	1.0	<LOR—0.9	<LOR		
lycopsamine	<LOR		<LOR—2.2	23	34	<LOR—105	14	24	<LOR—85	<LOR		
lycopsamine *N*-oxide	<LOR		<LOR—2.2	18	31	<LOR—111	<LOR		<LOR—2.7	<LOR		

^a^ Not resolved.

**Table 2:** HRAM data for pyrrolizidine alkaloids (or isomers) in *C. odorata*, tentatively identified using fragmentation patterns, with intermedine and intermedine *N*-oxide confirmed by comparison with the corresponding standard.

Alkaloid	Typical RT (min)	Molecular Ion Formula	Calculated [M + H]^+^	Observed [M + H]^+^ and Product Ions *m*/*z* (rel. abundance)
intermedine (**1**)	6.7	[C_15_H_25_NO_5_ + H]^+^	300.1806	300.1808 (5), 156.1021 (51), 139.0993 (11), 138.0915 (30), 120.0811 (18), 96.0813 (4), 95.0735 (4), 94.0656 (100), 82.0658 (5).
rinderine (**4**)	7.2	[C_15_H_25_NO_5_ + H]^+^	300.1806	300.1808 (5), 156.1021 (59), 139.0993 (16), 138.0915 (100), 120.0811 (27), 108.0812 (5), 96.0813 (20), 95.0735 (6), 94.0657 (21), 82.0658 (10)
rinderine *N*-oxide (**9**)	8.4	[C_15_H_25_NO_6_ + H]^+^	316.1755	316.1758 (30), 272.1495 (2), 226.1440 (3), 172.0970 (100), 155.0943 (18), 138.0916 (21), 136.0760 (7), 112.0761 (4), 111.0683 (10), 94.0657 (9), 93.0579 (7)
intermedine *N*-oxide (**6**)	8.7	[C_15_H_25_NO_6_ + H]^+^	316.1755	316.1758 (35), 272.1495 (2), 226.1439 (4), 172.0970 (100), 155.0943 (17), 138.0916 (55), 136.0760 (15), 111.0683 (12), 94.0657 (19), 93.0579 (14)

**Table 3:** HRAM data for pyrrolizidine alkaloids (or isomers) in *A. conyzoides*, tentatively identified by analysis of fragmentation patterns, with intermedine confirmed by comparison with the corresponding standard.

Alkaloid	Typical RT (min)	Molecular Ion Formula	Calculated [M + H]^+^	Observed [M + H]^+^ and Product Ions *m*/*z* (rel. abundance)
intermedine (**1**)	6.9	[C_15_H_25_NO_5_ + H]^+^	300.1806	300.1803 (5), 156.1019 (50), 138.0913 (31), 120.0809 (19), 95.0733 (4), 94.0655 (100)
rinderine (**4**)	7.4	[C_15_H_25_NO_5_ + H]^+^	300.1806	300.1805 (9), 156.1019 (77), 139.0992 (20), 138.0914 (100), 120.0809 (37), 96.0812 (19), 94.0656 (58)
rinderine *N*-oxide (**9**)	8.54	[C_15_H_25_NO_6_ + H]^+^	316.1755	316.1755 (29), 272.1488 (2), 226.1434 (3), 172.0966 (100), 155.0939 (19), 138.0912 (21), 136.0756 (7), 112.0758 (4), 111.0602 (2), 94.0655 (9), 93.0577 (7)
cynaustraline isomer 2	8.6	[C_15_H_27_NO_4_ + H]^+^	286.2013	286.2012 (28), 142.1227 (100), 125.1201 (8), 124.1124 (8)
intermedine *N*-oxide (**6**)	8.84	[C_15_H_25_NO_6_ + H]^+^	316.1755	316.1751 (34), 272.1487 (2), 226.1434 (4), 172.0967 (100), 155.0939 (17), 138.0913 (54), 136.0757 (15), 112.0759 (5), 111.0681 (11), 94.0655 (20), 93.0577 (14)
cynaustraline isomer	8.9	[C_15_H_27_NO_4_ + H]^+^	286.2013	286.2012 (34), 142.1227 (100), 124.1123 (62), 96.0812 (4)
7-*O*-angelylretronecine (minor)	8.94	[C_13_H_19_NO_3_ + H]^+^	238.1438	238.1442 (20), 138.0916 (35), 120.0810 (100), 108.0812 (58), 94.0656 (40), 83.0498 (26), 80.0500 (18).
further lycospamine *N*-oxide isomer	9.12	[C_15_H_25_NO_6_ + H]^+^	316.1755	316.1754 (42), 272.1494 (2), 226.1437 (4), 172.0968 (100), 155.0940 (23), 138.0914 (19), 136.0758 (24), 112.0760 (3), 111.0681 (6), 94.0656 (3), 93.0578 (6)
supinine (**15**)	9.2	[C_15_H_25_NO_4_ + H]^+^	284.1856	284.1853 (9), 140.1069 (73), 123.1043 (22), 122.0965 (100), 110.0966 (5), 94.0655 (9).
lycopsamine *N*-oxide	9.35	[C_15_H_25_NO_6_ + H]^+^	316.1755	316.1751 (41), 272.1491 (2), 226.1435 (3), 172.0966 (100), 155.0939 (17), 138.0913 (63), 136.0757 (17), 112.0758 (6), 111.0680 (11), 94.0655 (21), 93.0577 (16)
3′-O-acetylintermedine	9.5	[C_17_H_27_NO_5_ + H]^+^	342.1911	282.1690 (3), 156.1020 (24), 138.0910 (61), 120.0810 (36), 94.0656 (100),
9-*O*-angelylretronecine	9.8	[C_13_H_19_NO_3_ + H]^+^	238.1438	238.1439 (2), 138.0914 (11), 120.0810 (16), 96.0812 (5), 94.0656 (100), 83.0497 (5)
cynaustraline *N*-oxide isomer	9.8	[C_15_H_27_NO_5_ + H]^+^	302.1962	302.1961 (10), 158.1175 (100), 141.1148 (6), 140.1070 (3), 124.1122 (9)
supinine *N*-oxide (**16**)	10.1	[C_15_H_25_NO_5_ + H]^+^	300.1806	300.1084 (38), 156.1019 (100), 139.0992 (30), 138.0913 (6) 122.0965 (16), 121.0887 (10), 120.0809 (18), 108.0811 (4) 96.0812 (4)
amabaline *N*-oxide (**18**)	10.6	[C_15_H_25_NO_5_ + H]^+^	300.1806	300.1804 (42), 156.1019 (100), 139.0991 (30), 122.0965 (18), 121.0887 (11), 120.0809 (19), 108.0810 (4), 96.0811 (4)
3′-*O*-acetylsupinine (**23**)	10.9	[C_17_H_27_NO_5_ + H]^+^	326.1962	326.1951 (1), 266.1749 (8), 140.1069 (31), 123.1043 (15), 122.0965 (100), 120.0809 (4), 110.0967 (3), 94.0655 (12),
3′-*O*-acetylintermedine *N*-oxide	11.2	[C_17_H_27_NO_7_ + H]^+^	358.186	358.1859 (16), 316.1754 (4), 298.1648 (38), 172.0968 (100), 155.0940 (21), 138.0914 (71), 136.0758 (25), 111.0681 (18), 94.0656 (34), 93.0577 (21).
7-*O*-angelylretronecine *N*-oxide (minor)	11.2	[C_13_H_19_NO_4_ + H]^+^	254.1387	254.1386 (13), 172.0968 (19), 137.0836 (33), 136.0758 (16), 111.0682 (84), 106.0655 (100), 94.0656 (19), 83.0497 (23), 80 (28)
9-*O*-angelylretronecine *N*-oxide	11.9	[C_13_H_19_NO_4_ + H]^+^	254.1387	254.1385 (77), 193.1908 (11), 154.0862 (84), 138.0913 (90), 137.0835 (37), 136.0757 (98), 126.0914(92), 108.0810 (24), 94.0655 (47), 93.0577 (100), 83.0497 (65)
3′-*O*-acetylsupinine *N*-oxide (**24**)	12.1	[C_17_H_27_NO_5_ + H]^+^	342.1911	342.1908 (12), 300.1803 (3), 282.1697 (33), 156.1018 (100), 139.0991 (36), 122.0965 (28), 121.0887 (12), 120.0809 (30), 108.0810 (6), 96.0812 (7)
echimidine isomer (**25**)	13.8	[C_20_H_31_NO_7_ + H]^+^	398.2173	398.2173 (0), 138.0914 (6), 120.0810 (100), 108.0812 (2), 94.0656 (3), 93.0704 (3), 83.0496 (2), 55.0549 (1)
echimidine *N*-oxide isomer	15.2	[C_20_H_31_NO_8_ + H]^+^	414.2122	414.2122 (0), 396.2033 (8), 352.1734 (7), 254.1385 (71), 138.0914 (93), 137.08036 (100), 136.0757 (63), 120.0809 (72), 119.0732 (84), 111.0680 (31), 106.0655 (33), 94.0656 (86), 93.0577 (45), 83.0497 (20), 55.0550 (26)

**Table 4:** Tentatively identified pyrrolizidine alkaloids and the details used in the Orbitrap processing method, including formulae, retention times, precursor ions used for quantification and product ions used for confirmation.

Compound	Formula (M)	Typical R_T_ (min)	Precursor Ion (MH^+^) *m*/*z*	Product Ions (*m*/*z*)				
3′-*O*-acetylintermedine	C_17_H_27_NO_6_	9.5	342.1911	282.1705	156.1019	138.0913	120.0808	94.0651
3′-*O*-acetylintermedine *N*-oxide	C_17_H_27_NO_7_	11.2	358.1860	298.1649	172.0964	155.0941	138.0915	111.0682
3′-*O*-acetylsupinine	C_17_H_27_NO_5_	10.9	326.1962	266.1756	140.1075	122.0970	94.0656	
3′-*O*-acetylsupinine *N*-oxide	C_17_H_27_NO_6_	12.1	342.1911	282.1705	156.1025	139.0907	120.0810	
amabaline *N*-oxide	C_15_H_25_NO_5_	10.6	300.1805	156.1025	139.0991	122.0965	120.0810	
7-*O*-angelylretronecine	C_13_H_19_NO_3_	8.9	238.1438	138.0914	120.0810	108.0813	94.0653	
7-*O*-angelylretronecine *N*-oxide	C_13_H_19_NO_4_	11.2	254.1387	172.0968	137.0833	106.0655	94.0653	
9-*O*-angelylretronecine	C_13_H_19_NO_3_	9.8	238.1438	138.0914	120.0810	94.0653		
9-*O*-angelylretronecine *N*-oxide	C_13_H_19_NO_3_	11.9	254.1387	154.0862	138.0913	136.0757	93.0577	
cynaustraline isomer	C_15_H_27_NO_4_	8.9	286.2013	142.1226	124.1121			
cynaustraline *N*-oxide isomer	C_15_H_27_NO_5_	9.8	302.1962	158.1176	140.1070	124.1124		
echimidine isomer	C_20_H_31_NO_7_	13.8	398.2173	138.0914	120.0809	83.0497	55.0550	
echimidine *N*-oxide isomer	C_20_H_31_NO_8_	15.2	414.2122	254.1139	138.0914	137.0804		
rinderine	C_15_H_25_NO_5_	6.80	300.1806	156.1017	138.0914	120.0808	94.0655	
rinderine *N*-oxide	C_15_H_25_NO_6_	8.54	316.1755	172.0964	155.0937	138.0911	136.0755	94.0654
supinine	C_15_H_25_NO_4_	9.2	284.1856	140.1075	122.0970			
supinine *N*-oxide	C_15_H_25_NO_5_	10.1	300.1806	156.1025	138.0919			

**Table 5:** Stingless bee honey sample code and corresponding species name, country of origin, district area, together with LOR for each PA standard.

SBH Code	Species	Country	Region	LOR for Each PA Standard (ng/g)
GT-1	*Geniotrigona thoracica*	Malaysia	Selangor	5
GT-2			Selangor	5
GT-3			Selangor	5
GT-4			Selangor	5
GT-5			Selangor	0.75
HI-1	*Heterotrigona itama*	Malaysia	Sarawak	5
HI-2			Selangor	5
HI-3			Selangor	5
HI-4			Selangor	5
HI-5			Johor	5
HI-6			Selangor	5
HI-7			Selangor	5
HI-8			Kedah	5
HI-9			Selangor	5
HI-10			Selangor	5
TC-1	*Tetragonula carbonaria*	Australia	Brisbane	5
TC-2			Brisbane	0.75
TC-3			Brisbane	0.75
TC-4			Brisbane	0.75
TC-5			Brisbane	0.75
TC-6			Brisbane	5
TC-7			Brisbane	5
TC-8			Brisbane	n/a
TC-9			Brisbane	0.75
TC-10			Brisbane	0.75
TC-11			Brisbane	0.75
TH-1	*Tetragonula hockingsi*	Australia	Bundaberg	5
TH-2			Bundaberg	0.75
TH-3			Bundaberg	0.75
TH-4			Bundaberg	0.75
TH-5			Brisbane	0.75
TH-6			Brisbane	0.75
TH-7			Brisbane	0.75
TH-8			Brisbane	5
TH-9			Brisbane	0.75
TH-10			Brisbane	5

## Data Availability

The data supporting this study are provided within this article and its Supplementary Information.

## Supplementary Materials

The following supporting information can be downloaded at: https://www.mdpi.com/article/10.3390/toxins16010040/s1, Table S1: Pyrrolizidine alkaloids standards details used in the Orbitrap analysis of SBH and PA containing plants, including formulae, retention times, precursor ions used for quantitation and product ions used for confirmation.
