# Supplementary material for: Analysis of Pyrrolizidine Alkaloids in Stingless Bee Honey and Identification of a Botanical Source as Ageratum conyzoides
Source: Toxins (Basel). 2024 Jan 12;16(1):40. doi: 10.3390/toxins16010040 (PMC10819179; doi:10.3390/toxins16010040)
Supplement: Supplementary file 1 [file toxins-16-00040-s001.zip › toxins-2777298-supplementary.pdf]

Supplementary material: Analysis of Pyrrolizidine Alkaloids in Stingless Bee Honey and Identification of a Botanical Source as *Ageratum conyzoides*

Natasha L. Hungerford [1,*], Norhasnida Zawawi [1,2], Tianqi (Evonne) Zhu [1], Steve J. Carter [3], Kevin J. Melksham [3] and Mary T. Fletcher [1]

[1] Queensland Alliance for Agriculture and Food Innovation (QAAFI), The University of Queensland, Health and Food Sciences Precinct, Coopers Plains, QLD 4108, Australia; norhasnida@upm.edu.my or n.zawawi@uq.edu.au (N.Z.); mary.fletcher@uq.edu.au (M.T.F.)
[2] Faculty of Food Science and Technology, University Putra Malaysia, Serdang 43400, Malaysia
[3] Forensic and Scientific Services, Queensland Health, Coopers Plains, QLD 4108, Australia; steve.carter@health.qld.gov.au (S.J.C.); kevin.melksham@health.qld.gov.au (K.J.M.)
* Correspondence: n.hungerford@uq.edu.au

Table S1. Pyrrolizidine alkaloids standards details used in the Orbitrap analysis of SBH and PA containing plants, including formulae, retention times, precursor ions used for quantitation and product ions used for confirmation.

Compound	Formula (M)	Typical R_T (min)	Precursor Ion (MH^+) *m*/*z*	Product Ions (*m*/*z*)					
echimidine	$C_{20}H_{31}NO_7$	13.37	398.2173	120.0809	83.0497	55.0550			
echimidine *N*-oxide	$C_{20}H_{31}NO_8$	13.39	414.2122	396.2004	352.1745	254.1379	220.1326	137.0833	
erucifoline	$C_{18}H_{23}NO_6$	6.57	350.1598	322.1642	220.1329	164.1066	138.0911	120.0807	
erucifoline *N*-oxide	$C_{18}H_{23}NO_7$	8.17	366.1547	278.1386	218.1172	164.1067	136.0756	119.0729	
europine	$C_{16}H_{27}NO_6$	7.29	330.1911	254.1385	156.1019	138.0914	120.0810	96.0812	
europine *N*-oxide	$C_{16}H_{27}NO_7$	8.12	346.1860	328.1743	270.1328	256.1172	172.0964	155.0937	
helioamplexine[a]	$C_{16}H_{27}NO_5$	9.61	314.1962	156.1017	138.0913	120.0808	94.0655		
helioamplexine *N*-oxide[a]	$C_{16}H_{27}NO_6$	10.67	330.1911	172.0966	155.0938	138.0913	111.0913	94.0653	
heliotrine	$C_{16}H_{27}NO_5$	9.97	314.1962	156.1017	138.0913	120.0808	94.0655		
heliotrine *N*-oxide	$C_{16}H_{27}NO_6$	11.02	330.1911	172.0966	155.0938	138.0913	111.0913	94.0653	
indicine	$C_{15}H_{25}NO_5$	7.09	300.1806	156.1019	138.0914	120.0810	94.0656	82.0657	
indicine *N*-oxide and intermedine *N*-oxide (n.r.)[b]	$C_{15}H_{25}NO_6$	8.66	316.1755	226.1437	172.0968	155.0941	138.0914	111.0682	94.0656
intermedine	$C_{15}H_{25}NO_5$	6.73	300.1806	210.1488	156.1019	138.0914	120.0810	94.0656	
jacobine	$C_{18}H_{25}NO_6$	6.98	352.1755	308.1485	280.1539	262.1432	234.1483	155.1063	
jacobine *N*-oxide	$C_{18}H_{25}NO_7$	8.2	368.1704	296.1485	190.1222	139.0989	121.0885	120.0807	
lasiocarpine	$C_{21}H_{33}NO_7$	15.53	412.2330	238.1435	156.1020	138.0914	120.0810	94.0656	
lasiocarpine *N*-oxide	$C_{21}H_{33}NO_8$	16.75	428.2279	410.2168	352.1746	328.1753	254.1384	220.1333	137.0835
lycopsamine	$C_{15}H_{25}NO_5$	7.25	300.1806	156.1017	138.0914	120.0808	94.0655		

Compound	Formula (M)	Typical R_T (min)	Precursor Ion (MH^+) *m*/*z*	Product Ions (*m*/*z*)				
lycopsamine *N*-oxide	$C_{15}H_{25}NO_6$	9.16	316.1755	172.0964	155.0937	138.0911	136.0755	94.0654
monocrotaline	$C_{16}H_{23}NO_6$	3.35	326.1598	280.1548	237.1354			
monocrotaline *N*-oxide	$C_{16}H_{23}NO_7$	7.87	342.1547	314.1590	296.1487	236.1274	137.0833	119.0729
retrorsine	$C_{18}H_{25}NO_6$	9.64	352.1755	324.1802	138.0913	120.0808	94.0655	
retrorsine *N*-oxide	$C_{18}H_{25}NO_7$	10.15	368.1704	220.1340	154.0862			
senecionine	$C_{18}H_{25}NO_5$	11.74	336.1806	308.1864	120.0809			
senecionine *N*-oxide	$C_{18}H_{25}NO_6$	12.41	352.1755	324.1825	220.1332			
seneciphylline	$C_{18}H_{23}NO_5$	10.08	334.1649	306.1706	120.0811			
seneciphylline *N*-oxide	$C_{18}H_{23}NO_6$	10.97	350.1598	322.1656	246.1495			
senecivernine	$C_{18}H_{25}NO_5$	11.43	336.1806	308.1848	153.0907	138.0911	120.0807	94.0654
senecivernine *N*-oxide	$C_{18}H_{25}NO_6$	11.98	352.1755	324.1795	220.1327	154.0859	136.0755	120.0807
senkirkine	$C_{19}H_{27}NO_6$	14.47	366.1911	168.1020	150.0915			
trichodesmine	$C_{18}H_{27}NO_6$	9.34	354.1911	308.1857	223.1203	222.1489	164.1071	121.0889

[a] Standard isolated from *Heliotropium amplexicaule* and confirmed by NMR to be >95% [19]. [b]n.r. = not resolved.
